# Machine learning clinical decision support systems for surveillance: a case study on pertussis and RSV in children

**DOI:** 10.3389/fped.2023.1112074

**Published:** 2023-05-22

**Authors:** Kimberly A. Mc Cord—De Iaco, Francesco Gesualdo, Elisabetta Pandolfi, Ileana Croci, Alberto Eugenio Tozzi

**Affiliations:** Predictive and Preventive Medicine Research Unit, Bambino Gesù Children's Hospital IRCCS, Rome, Italy

**Keywords:** machine learning, artificial intelligence, surveillance, clinical decision support systems, pertussis, respiratory syncytial virus, infectious diseases

## Abstract

We tested the performance of a machine learning (ML) algorithm based on signs and symptoms for the diagnosis of RSV infection or pertussis in the first year of age to support clinical decisions and provide timely data for public health surveillance. We used data from a retrospective case series of children in the first year of life investigated for acute respiratory infections in the emergency room from 2015 to 2020. We collected data from PCR laboratory tests for confirming pertussis or RSV infection, clinical symptoms, and routine blood testing results, which were used for the algorithm development. We used a LightGBM model to develop 2 sets of models for predicting pertussis and RSV infection: for each type of infection, we developed one model trained with the combination of clinical symptoms and results from routine blood test (white blood cell count, lymphocyte fraction and C-reactive protein), and one with symptoms only. All analyses were performed using Python 3.7.4 with Shapley values (Shap values) visualization package for predictor visualization. The performance of the models was assessed through confusion matrices. The models were developed on a dataset of 599 children. The recall for the pertussis model combining symptoms and routine laboratory tests was 0.72, and 0.74 with clinical symptoms only. For RSV infection, recall was 0.68 with clinical symptoms and laboratory tests and 0.71 with clinical symptoms only. The F1 score for the pertussis model was 0.72 in both models, and, for RSV infection, it was 0.69 and 0.75. ML models can support the diagnosis and surveillance of infectious diseases such as pertussis or RSV infection in children based on common symptoms and laboratory tests. ML-based clinical decision support systems may be developed in the future in large networks to create accurate tools for clinical support and public health surveillance.

## Introduction

Public health strategies for controlling and preventing infectious diseases are based on surveillance to continuously collect, analyze, and interpret data from the healthcare system (1). As surveillance systems should ideally collect data from different settings, case definitions are used to standardize the detection and monitoring of events to allow for trend interpretation (2). Specificity of case definitions depends on the combination of clinical symptoms and on the use of laboratory tests for confirmation (3). Clinical case definitions are valuable at the point of hospital care for rapid triage, for determining the need of laboratory tests, and for deciding the most appropriate management and treatment (2). Syndromic surveillance has been developed in response to emerging threats to timely recognize unexpected patterns of symptoms (4), and has been extensively tested for influenza (5). However, there is much less effort on extending syndromic surveillance to other diseases and to settings where a timely management of cases is needed and where laboratory facilities are not immediately available.

Pertussis is an endemic and highly transmissible disease occurring worldwide (6). Although efficacious vaccines are routinely used for controlling the disease and high immunization coverage rates have been achieved over time, *B. pertussis* has continued to circulate, possibly due to waning immunity after immunization and natural infection (6). Respiratory Syncytial Virus (RSV) is extremely common: it is estimated that almost every child experiences an infection by RSV by the age of 2 (7, 8). The epidemiology of pertussis and RSV infection is traditionally seasonal, with peaks of pertussis in spring-summer and peaks of RSV infections during autumn-winter (9, 10). Despite their distinct etiology, pertussis and RSV infections in infants may be a diagnostic challenge for clinicians due to similar clinical features (11, 12). The increased availability of laboratory tests for confirming the etiology of these infections has been helpful to counteract the low specificity of respiratory symptoms.

The epidemiology of acute respiratory infections, including pertussis and RSV infection (12, 13), has been disrupted by the SARS-CoV-2 pandemic. Social distancing policies, face mask wearing and possibly some ecological pressure exerted by SARS-CoV2 have contributed to a decrease in the circulation of other respiratory pathogens (14). After the easing of containment policies, respiratory infections other than COVID-19 were expected to re-emerge (12).

Artificial intelligence (AI) has been indicated as a powerful tool for improving the timeliness and accuracy in surveillance systems gathering information from heterogeneous sources (15). There is also the need to move towards pre-syndromic surveillance, to identify space-time clusters of novel syndromic entities not yet recognised by traditional systems, which may be detected with the support of AI-based techniques (16, 17). In essence, AI algorithms developed for syndromic surveillance on large datasets may be more accurate and flexible than traditional case definitions.

Clinical decision support systems (CDSS) based on AI models provide physicians with patient-specific assessments or recommendations towards a decision tailored on the specific characteristics of each patient and based on current evidence (18). In a post-COVID scenario, developing such systems to provide diagnosis support for acute respiratory diseases before laboratory confirmation may have important public health and clinical implications.

As clinical case definitions for some respiratory infections currently have a low specificity, we focused on the hypothesis that AI algorithms may be trained to recognize specific infections to support clinical decisions and public health surveillance. For these reasons, we tested the performance of a ML algorithm for the diagnosis of RSV infection or pertussis in the first year of age at the emergency department, and developed models suitable for different settings.

## Methods

### Population and settings

Italy has a universal, free of charge healthcare system, which includes primary pediatric care for all children. Family pediatricians are available for pediatric visits during office hours. Clinical requests outside office hours or holidays and urgent clinical problems are usually referred to Emergency Rooms, where services are offered continuously (19).

The population included in this study consisted of patients enrolled in previous surveillance studies coordinated by the Bambino Gesù Children's Hospital, a pediatric research hospital located in Rome, Italy (20). In previous surveillance activities, we enrolled children less than 1 year of age presenting to the emergency department with respiratory symptoms between August 2015 and June 2020 and, after obtaining a signed informed consent, a research nurse collected, through a standardized questionnaire, demographic information and respiratory signs and symptoms. For each patient, we also recorded, through patients' hospital records, full white blood cell count, lymphocyte fraction and the result of a multiplex PCR test on nasopharyngeal aspirates for respiratory pathogens, including *B. pertussis* and RSV. We did not include asymptomatic patients or those with other symptoms. Fever was defined as follows: single > 37.8°C oral/tympanic membrane (21). To evaluate hypoxia, we considered the arterial oxygen saturation (SaO2) [which refers to the amount of oxygen bound to hemoglobin in arterial blood]. The measurement is given as a percentage. Resting SaO2 less than or equal to 95% was considered abnormal. “Cough” and “paroxysmal cough” were treated as two independent variables. This is consistent with the clinical observations in the clinic, where these two symptoms may be observed independently. Only one child had a paroxysmal cough but no “normal” cough (i.e., most patients experiencing paroxysmal coughs also report “normal” cough).

### Laboratory confirmation

Nasopharyngeal aspirates were collected and processed using a standardized protocol (22).

The presence of *B. pertussis* was investigated using a Bordetella Real Time PCR kit targeting IS481 (Bordetella R-gene assay Argene, Biomerieux, Marcy l'Etoile, France). For RSV identification, we used a commercial multiplex RT-PCR kit (AllplexTM Respiratory Full Panel Assay) for 16 respiratory viruses including RSV A and B, influenza virus A and B, human coronavirus OC43, 229E, NL-63 and HUK1, adenovirus, human rhinovirus, parainfluenza virus 1–2–3–4, human metapneumovirus and human bocavirus.

### Feature selection

The diagnostic outcome for pertussis and RSV infection data was already labeled, and there was no missing data for this variable. We defined a binary outcome (normal or high) for three laboratory values based on standard pediatric cutoffs, as follows. For C-reactive protein (CRP), any value greater than 1.0 ml/dl was considered high. White blood counts were considered high if they were greater than 34.0 × 103/*μ*l at 30 days of age and 19.5 × 103/μl over 30 days of age (23). The lymphocyte fraction was considered high if greater than 50% at any age. Symptoms recorded for this study included Cough, Cyanosis, Emesis, Petechiae, Paroxysmal cough, Fever, Conjunctival petechiae, Stridor, Hypoxia, Dehydration, Convulsions, Difficulty feeding and Pre-existing conditions, which were also classified as binary variables.

### Machine learning approach

We performed a retrospective binary classification to identify patients diagnosed with pertussis (model 1) or RSV infection (model 2) with a set of symptoms, either with (model 1–2 a) or without (model 1–2 b) routine blood testing results (CRP, white blood cell count, lymphocyte fraction). Rather than developing a complex model to identify the possible respiratory pathogen, we focused on developing simple models to discern between the 2 pathogens most likely to require additional interventions or early treatment in clinical care. For pertussis, the aim was to identify patients early in order to initiate antibiotic treatment as soon as possible; whereas for RSV infection the aim was to identify patients with this viral illness in order to exclude other respiratory pathogens and orient proper management.

Since different clinical sites may have different needs and resources, we developed 4 models so that we could 1) identify patients presenting with pertussis a) using clinical symptoms and routine blood testing, or b) simply using clinical symptoms; or 2) to identify RSV infection a) using clinical symptoms and routine blood testing, or b) simply using clinical symptoms.

To enable estimation of pertussis and RSV infection case predictions with minimal clinical data, we conducted a retrospective diagnostic classification analysis by training gradient-boosting decision tree binary classifiers. We compared several ML models (Logistic Regression, Gaussian Naive Bayes, Random Forest and Light Gradient Boosting Machine—LightGBM) before choosing LightGBM as the highest performing at a moderate time cost as well as being able to ingest missing values (which may be useful for point-of-care CDSS implementation). All analyses were performed using Python 3.7.4 with Shapley values (Shap values) visualization package for predictor visualization.

In order to reduce the complexity of the models as much as possible, we included 14 binary clinical symptoms (Cough, Cyanosis, Emesis, Petechiae, Paroxysmal cough, Fever, Conjunctival petechiae, Stridor, Hypoxia, Dehydration, Convulsions, Difficulty feeding and Pre-existing conditions) and four demographic binary features (gender male/female, age less than/greater than 30 days, prematurity yes/no, duration of illness greater than/less than 3 days) in the basic models (models b) and included the most commonly performed testing for emergency departments or clinics in the primary model (lymphocyte count high/normal, white blood cell count high/normal, CRP high/normal—models a).

While there was a significant class imbalance, we did not wish to introduce bias and thus avoided resampling the data. Due to the class imbalance and the nature of the application of the model, we focused on the recall metric for model selection and hyperparameter tuning (as to minimize false negatives).

Hyperparameter tuning of each LightGBM model was performed using the verstack package (Optuna) and feature importance graphs were obtained with the Shap package.

## Results

### Data and patient characteristics

The sample size was 599 children, but 14 observations were dropped since children were positive to both *B. pertussis* and RSV. Out of 585 final observations, 154 (26%) patients had a positive PCR test for *B. pertussis*, 97 (17%) had a positive PCR test for RSV, in 123 patients (21.0%) the RT-PCR was not able to identify a pathogen among the 16 included in the diagnostic panel, while the remaining patients with respiratory symptoms had evidence of other viral infections. 313 (54%) patients were males and 272 (46%) females, 514 (88%) were over a month of age and 71 (12%) were less than 30 days old at diagnosis (median age in days 63, IQR 42–108 days). The symptom duration was less than or equal to 3 days in 272 children (46%) and greater than 3 days in 313 (54%) children (median duration in days 9, IQR 2–11). A total of 179 children (14%) were born preterm (less than 37 weeks of gestation). White blood cell count was normal in 508 (87%) children, whereas lymphocyte fraction was elevated in 318 (54%). The frequency of symptoms was the following: cough 85%, paroxysmal cough 56%, emesis 39%, fever 38%, cyanosis 34%, stridor 25%, hypoxia 11%,difficulty feeding 11%, petechiae 5%, conjunctival petechiae 5%, dehydration 2% and convulsions 1%. Fifty-six children (10%) had pre-existing conditions, mostly chronic diseases.

### Model performance

The performance of our four ML models was fair (Table 1). For pertussis (model 1) recall was 0.72 including laboratory testing and 0.74 with clinical symptoms only. For RSV infection (model 2), recall was 0.68 including laboratory testing and 0.71 with clinical symptoms only. The F1 score for the pertussis model was 0.72 in both models a and b, and, for RSV infection, it was 0.69 and 0.75 for models a and b, respectively. The area under the receiver operating characteristic curve (AUC) was 0.69 for model 1a, 0.71 for model 1b, 0.59 for model 2a, and 0.62 for model 2b.

**Table 1:** Model performance.

	Pertussis (model 1)		RSV (model 2)	
	Model A	Model B	Model A	Model B
Recall	0.72	0.74	0.68	0.71
1 class	0.61	0.72	0.67	0.67
0 class	0.83	0.75	0.69	0.75
Precision	0.72	0.71	0.60	0.62
F1	0.72	0.72	0.59	0.63
AUC	0.69	0.71	0.59	0.62

Model A refers to the main model including basic laboratory data (hospital/clinic), model B refers to the alternative model including only syndromic data (for outpatient settings without availability of performing laboratory data).

**Figure 1:**
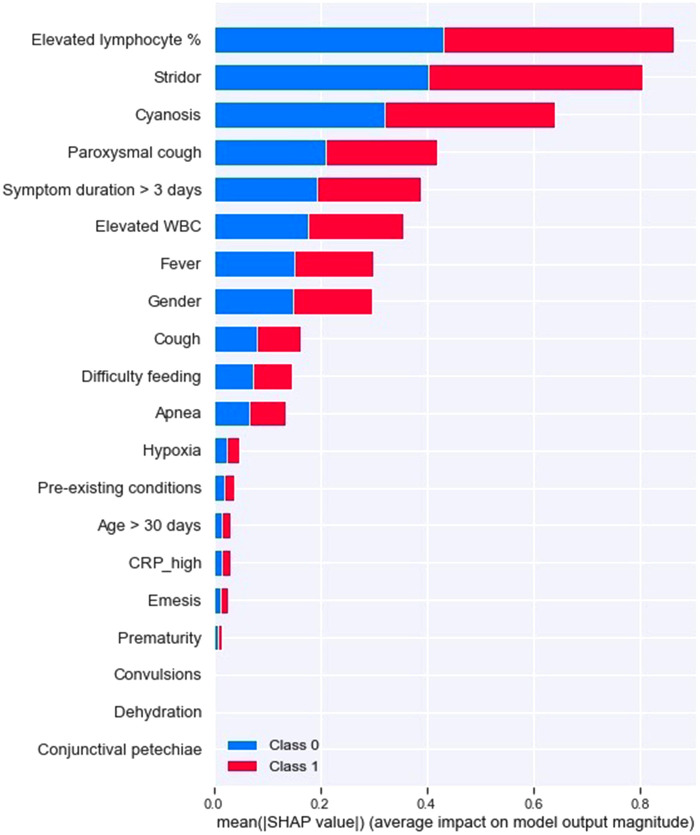
Pertussis model A (with laboratory testing).

**Figure 2:**
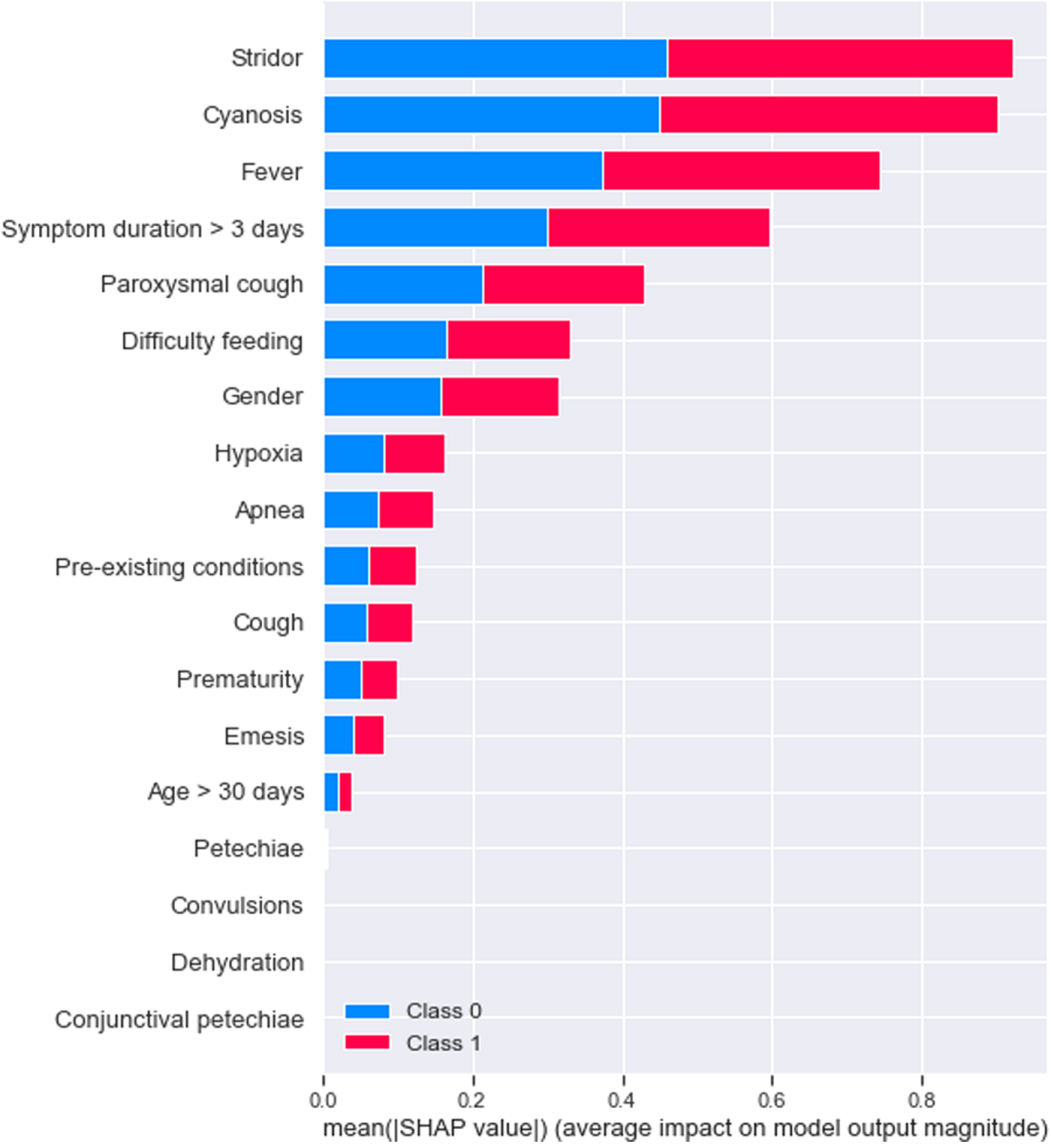
Pertussis model B (syndromic only).

**Figure 3:**
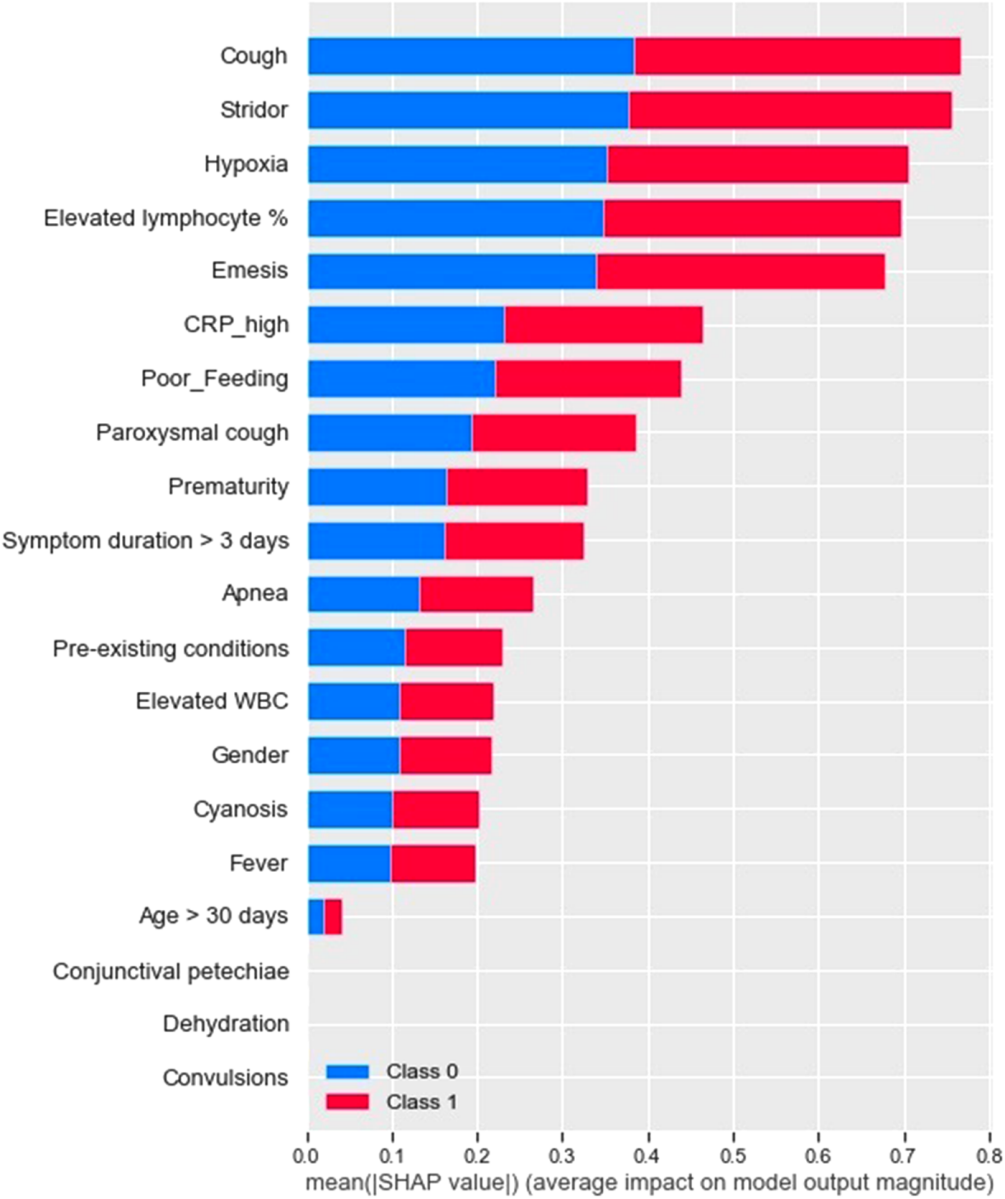
RSV model A (with laboratory testing).

**Figure 4:**
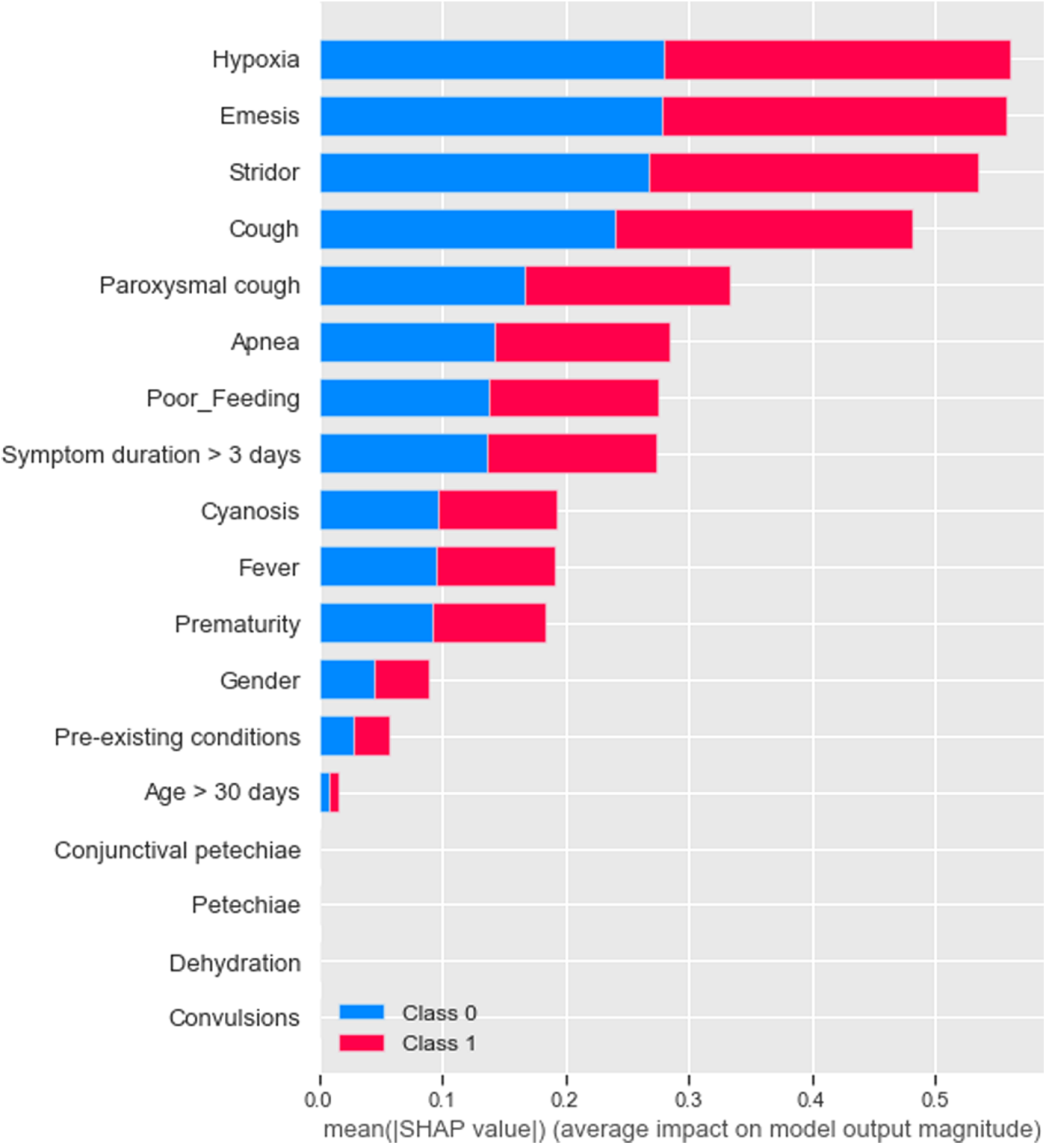
RSV model B (syndromic only).

For pertussis model 1a, the principal driving feature was elevated lymphocyte fraction, followed by stridor and cyanosis (Figure 1); whereas for pertussis model 1b, the most important features were stridor, cyanosis and absence of fever (Figure 2). For RSV model 2a the most important features were cough, stridor and hypoxia (Figure 3); and for RSV model 2b they were hypoxia, emesis and stridor (Figure 4). Most of the feature's Shap values contributed fairly equally among the two classes.

Overall the model which performed the best was the pertussis prediction without the inclusion of laboratory testing, and for both illnesses the addition of these three features did not improve the prediction in a substantial way. Generally, negative patients to both infections had better prediction scores (hence it was easier for the model to predict children who did not have pertussis or RSV infection than those that were sick), likely due to the class imbalance. We only had 4.5% missing data. Due to the nature of the data collection (prospective, active, by research nurses) we didn't feel comfortable assuming that missing data would equate to a “0” (symptom not present, which would be a safe assumption in a clinical note). Hence, these rows were dropped to avoid imputation and potential addition of bias to the analysis.

### Confusion matrices

The confusion matrices are reported in Table 2. Briefly, the sensitivity and specificity were 83% and 61% for pertussis model a and 75% and 72% for model b. For the RSV model, the sensitivity was 69% and the specificity 67% for model a, and 74% and 69% for model b.

**Table 2:** Confusion matrix.

	Pertussis (model 1)		RSV (model 2)	
	Model A	Model B	Model A	Model B
Sensitivity	0.83	0.75	0.69	0.74
Specificity	0.61	0.72	0.67	0.66
Total test set (*n*)	117	117	117	117
True positives (*n*)	22	26	12	12
True negatives (*n*)	67	61	68	74
False positives (*n*)	14	20	31	25
False negatives (*n*)	14	10	6	6

Model A refers to the main model including basic laboratory data (hospital/clinic), model B refers to the alternative model including only syndromic data (for outpatient settings without availability of performing laboratory data).

Accordingly, in our holdout set of 117 children, we would have missed 14 cases of pertussis and classified another 14 with an incorrect pertussis diagnosis (model 1a). For RSV infection, we would have missed only six children with the virus but would have incorrectly diagnosed 31 with having the virus when in fact they were negative. Depending on the preference of the hospital or pediatrician's office, the standard threshold of 0.5 (where any predicted probability above would be classified as a 1 and inversely as a 0) could be adjusted to further reduce false positives or false negatives.

## Discussion

We developed four models for the diagnosis of pertussis and RSV infection that, based on clinical symptoms and routine blood tests, accurately predict the laboratory test confirmation for these pathogens. These models represent a prototype which could be deployed in an application (eg. for smartphones or for electronic health records) that may be integrated in routine clinical care to assist clinicians in the proper management of these diseases and to provide information that may be immediately available for public surveillance. To our knowledge, no Machine Learning-Clinical Decision Support Systems (ML-CDSS) exists yet to support the diagnosis of pertussis or RSV infection in children.

Our work suggests that AI-based decision support systems may be developed with little clinical data to identify pertussis and RSV infection in children < 1 year of age. The same approach may be easily applied to other infectious diseases that would benefit from surveillance.

Interestingly, the performance of the two models was not improved by the addition of routine blood tests. Regarding pertussis, there was a slight improvement in the detection of the cases with the inclusion of laboratory testing in terms of recall performance, which is consistent with the clinical practice of considering lymphocytosis for the differentiation of pertussis vs. other respiratory infections. However, the overall model performance was not strong enough to justify its deployment, considering that classifying patients with symptoms features only is less complex and more scalable.

The models for pertussis yielded a higher classification performance than those for RSV infection, however, the sample size was lower for RSV infection and the class imbalance more pronounced, therefore, it is possible that the performance could improve basing the analysis on a larger dataset. While these predictions alone may not replace a laboratory test for confirmation, a CDSS integrating these models at the point of care could help to timely support the clinician's judgment in further testing or initiating early treatment when necessary. The simplicity of a CDSS based only on syndromic data makes it invaluable during periods of changing epidemiology, such as the one we are facing with RSV infections in the aftermath of COVID-19, providing information immediately suitable for public health surveillance.

Indeed, other studies attempted COVID-19 case predictions based on AI algorithms (24), but required additional sources of information, such as data obtained from patients through surveys, possibly leading to delays as well as selection biases, which would be minimized with a system embedded at the clinical point-of-care.

A further development of this system could consist in a model for identification of novel clinical syndromes. This kind of model should be fed with unlabelled data collected prospectively and should be based on unsupervised learning.

In a hypothetical future scenario, the identification of individual cases of infectious diseases within the community in a rapid timeframe, as well as the ability to flag novel syndromes or pathogens that pose a threat to public health systems, will rely on fully automated and integrated systems across hospitals and primary care providers, similar to the one conceptualized in this study.

We chose two case studies that have relevance both from the point of view of the clinician and for public health. A pertussis clinical case definition for surveillance is available, although it is not very helpful at the point of care, as it includes a long duration of cough that may only be assessed after early medical encounters (19). We already demonstrated that a decision tree algorithm outperforms the accuracy of a clinical case definition for pertussis (19).

The interest for RSV surveillance has recently increased, as new candidates of RSV vaccines and monoclonal antibodies are in late-stage clinical trials (25–27). However, a universal surveillance system for RSV does not exist yet. A standard algorithmic approach may be scaled up to multiple institutions and support timely surveillance systems.

Indeed, syndromic surveillance at the emergency department has been frequently used to complement existing surveillance systems (5) and may remain in place for a long time (28, 29). These systems have been classically developed for monitoring emerging threats from bioterrorism (30).

ML-CDSS for infectious diseases are drawing increased interest, but a minority of them have been developed in emergency care or primary care settings (31). A recent review showed that ML-CDSS for diagnosis have been developed for tuberculosis, healthcare associated infections, surgical site infections, infections at admission into hospital, bacterial or viral meningitis (31).

The next steps for this pilot would be the deployment of our algorithms in an EHR system, making predictions but not displaying them to the physicians (shadow mode). This way, we could manually validate a subsample of these predictions and compare them to a gold standard (such as a PCR result). Only then would we release the AI algorithm monitoring changes in clinical performance (quality control study).

The importance of our work is in representing a proof of concept of ML-CDSS for diagnosis of infectious diseases that can be collectively developed and trained by multiple sites increasing performance and generalizability. These systems may be deployed either into electronic health records and in simple applications for smartphones to enhance or create sustainable and timely surveillance systems for events of interest in hospital and primary care settings.

### Limitations

One of the main limitations of our study is its retrospective nature. Our model is based on data collected for two previous surveillance studies on respiratory infections. Therefore, although the quality of our data is high, the data collection had not been specifically designed with the purpose of developing a ML-CDSS. An improvement of the model could be achieved through a prospective study design, which would enable us to collect additional data to improve the diagnostic evaluation and increase the accuracy of the model. Additional clinical information that might be considered for a future model are respiratory rate, chest retractions and breath sounds (rales, wheezing), which are typically found in children with RSV infections. An improved model to be applied in high resource settings might also include audio tracks of respiratory auscultation.

Another limitation concerns the fact that we transformed continuous variables (such as oxygen saturation, fever, leukocyte/lymphocyte counts, CRP) into binary variables using cut-off values based on the published literature. A possible improvement of the model should include an assessment of the variability in the accuracy based on different thresholds of the continuous variables.

Another limitation of our analysis is that it was difficult to determine if the predictive abilities of our models were magnified or minimized by our data. On the one hand, since our data was collected in the emergency room, there likely was a selection of more severe (and possibly easier to identify) patients. On the other hand, the relatively small sample size, particularly with class imbalance, may have underestimated the real signal. However, our example can be improved with additional data from multiple sites and serves the purpose of describing the potential of a CDSS in the Italian healthcare system.

Another limitation regards the elimination of missing data. We decided to adopt a conservative approach, and to drop the records that included missing data. Although this may have biased the result, the proportion of missing data was very small (4.5%), therefore we do not expect it to have had a major impact on our results.

Moreover, we decided not to include observations with coinfections in our model, in order to minimize the influence of coinfections on our findings. The number of coinfections in our dataset was actually small (2.3%), but the fact that they were not included in the model might limit its applicability. A potential amelioration of our model should better take into account the possibility of having patients with coinfections.

The high rate of false positive for RSV yielded by our model may seem worrying. However, this may suggest that such tools may be more helpful in epidemic settings, when the prevalence of the condition is high. Again, a better performance of the model may be achieved including a larger set of parameters in the model, as previously discussed.

Finally, generalizability could be also limited by the high quality of our data, due to the research setting in which it was gathered. Validation of the model in a wider, real world scenario would better elucidate its potentials.

## Conclusions

ML-CDSS may represent valuable tools for surveillance of infectious diseases. We present here a proof-of-concept which could serve as a basis for the future development of AI-based surveillance systems. The model we tested in this feasibility study included a limited set of high-quality clinical and laboratory parameters, obtained in a specific research setting, which might limit its performance and generalizability. To improve performance and generalizability, future research steps should aim at a collective effort to: enlarge the dataset used for training and validation, including data from different settings; increase number of parameters used to train and validate the model; testing the model in a real life scenario, e.g., shadow testing on EHRs. These perspectives deserve to be further explored to extend the application of artificial intelligence to public health surveillance.

## Data Availability

The datasets presented in this article are not readily available due to institutional constraints. Requests to access the datasets should be directed to Alberto Eugenio Tozzi, albertoeugenio.tozzi@opbg.net.
